# Osteo-Plastic Operations

**Published:** 1860-04

**Authors:** 


### EDITORIAL DEPARTMENT.

—The paper which we publish this month upon *Osteo-Plastic Operations*, from the pen of one of the most distinguished surgeons of the age, Prof. Langenbeck, of Berlin, is upon a subject of the very highest importance. The recent researches of M. Ollier upon "The Artificial Production of Bone, by means of the transplantation of the periosteum, and the regeneration of bone after exsection, and complete removal," which have been doubly crowned by the Academy of Sciences and the Academy of Medicine, have opened a new avenue for surgical exploits. The report of the several successful operations given in this paper is the legitimate result of the experimental researches made by M. Ollier. The application to man of the physiological data derived from experiments upon animals, is a brilliant suggestion in the direction of conservative surgery, and will tend to lead surgeons still further towards the preservation of form, in operations requiring extensive mutilation, such as was formerly the case in operations similar to those related in the paper given in the present number of the MONTHLY.

In this connection, we may state that M. Ollier has added another experiment in relation to the transplantation of bone, to those already given in his Essay, which appeared in Brown-Séquard's *Journal de la Physiologie*.

M. Sedillot, of Strasbourg, in a note addressed to the Academy of Sciences in December last, denied that there was any well-authenticated case of complete osseous regeneration under the periosteum, sufficiently produced to replace the ancient bone and fulfill its functions.

In reply to this, M. Ollier cited, in a subsequent sitting, several cases drawn from the practice of experienced surgeons, to sustain his views, showing that the movements were preserved, and the various functions of the original bone maintained. At a still more recent sitting, M. Ollier contributed a note upon the transplantation of bone taken from animals dead a certain length of time, which goes to show still more forcibly the remarkable aptitude which these tissues have to unite. "Not only has he transplanted entire bones immediately from the midst of the tissues of a living animal, but he has succeeded in reviving them upon another animal of the same species, although the animal from which they were taken had been dead some little time." "The vitality of these tissues," says M. Ollier in this note, "is not extinguished with the circulation and respiration; transplanted into a situation analogous to that they formerly occupied, they continue to live and to grow to a certain degree, according to the laws of their normal development."

" Portions of periosteum taken from rabbits which have died either from hæmorrhage, or from a section of the medulla oblongata, were engrafted, and gave osseous productions ten, thirty, sixty and eighty minutes after the heart had ceased to beat. Entire bones, (humerus, tibia, radius, &c.,) transplanted ten, thirty and sixty minutes after death, united perfectly. In these different experiments the union was real; since the transplanted bones presented, at the end of five months, the following characteristics: They were perfectly adherent to the tissues in which they had been placed; they were covered with a sub-periosteal osseous layer of new formation; they were permeable to injections thrown in by the arteries."

M. Forget, in reviewing the subject in the *Union Médicale*, for February 21, thinks that the formula laid down by M. Flourens, to the effect that, "preserving the periosteum upon removing a bone, the preserved periosteum will *produce bone*," should be so modified as to read " will produce bony tissue." This is quite sufficient for all surgical purposes, and until experiment proves demonstrably the aphorism of M. Flourens to be correct, we shall be content to accept the modification, not regarding it as in any way interfering with the great practical results to be drawn from it, for the use of the bold surgeon.

